# Exercise interveNtion outdoor proJect in the cOmmunitY – results from the ENJOY program for independence in dementia: a feasibility pilot randomised controlled trial

**DOI:** 10.1186/s12877-023-04132-5

**Published:** 2023-07-12

**Authors:** Pazit Levinger, Anita M. Y. Goh, Jeremy Dunn, Josephine Katite, Ritu Paudel, Adrian Onofrio, Frances Batchelor, Maya G. Panisset, Keith D. Hill

**Affiliations:** 1 National Ageing Research Institute, Royal Melbourne Hospital, PO Box 2127, Melbourne, Victoria 3050 Australia; 2 Institute for Health and Sport, Victoria University, Melbourne, Australia; 3 Rehabilitation, Ageing and Independent Living (RAIL) Research Centre, Monash University, Melbourne, Australia; 4 The University of Melbourne, Melbourne, Australia; 5 Abound Communities, Melbourne, Australia

**Keywords:** Dementia, Cognitive decline, Physical activity, Falls, Built environment, Age-friendly, Seniors Exercise Park

## Abstract

The Seniors Exercise Park program is an evidence-based outdoor physical and social activity program designed originally for older people with no cognitive impairment. This study aimed to pilot this program for people living with dementia in residential aged care. We examined the feasibility of delivering the program, evaluating its structure, safety, and supervision needs. In addition, physical, social, health and cognitive benefits of participation were examined.

**Method**

This was a feasibility pilot randomised controlled design. Adults aged ≥ 60 years with symptoms of dementia and/or diagnoses of dementia were recruited from an aged care facility in Australia. Participants allocated to the intervention underwent a 12-week structured supervised physical activity program using the outdoor Seniors Exercise Park equipment followed by a 12-week maintenance phase, while the controls received usual care programs. Assessments occurred at baseline, 12 and 24-weeks. Feasibility evaluation included recruitment rate, retention, attendance, overall adherence, dropout rate, adverse events, program delivery modifications and supervision requirements. A suite of cognitive and health-related questionnaires and physical function measures were also collected.

**Results**

Sixteen participants were recruited (recruitment rate: 58.6%), eight for the intervention (83.3 ± 7.5 years, 87.5% women) and eight for the control (age 87.5 ± 3.0 years, 87.5% women). Eighty-eight percent completed the 12-week structured program, with 75% retention at 24-weeks. Across the 24-week period, 84.3% participation adherence was reported. No falls or adverse events occurred. Modifications of the program mainly related to method of communication, cueing and adjustments to suit individual personality and characteristics. A ratio of one trainer to two participants was practical and safe. There were no significant changes over time between groups in any of the secondary outcomes. High level of engagement, enjoyment and mood was reported throughout the exercise program.

**Conclusion**

The Seniors Exercise Park physical activity program was safe and feasible for people living with dementia in residential care, with high levels of enjoyment, positive attitude, and engagement reported in the intervention group. Individualised communication during program delivery was needed to facilitate motivation and participation. Further research is needed to assess the program effectiveness on physical and cognitive function on a larger scale.

**Trial registration**

This trial is registered with the Australian New Zealand Clinical Trials Registry—Registry Number ACTRN12620000733976. Registered on the 13/07/2020.

## Introduction

Around 55 million people were reported to have dementia worldwide in 2020 [1]. With the expected increase in the aging population, this number is expected to rise to 78 million in 2030 and 139 million in 2050 [1]. In Australia, it was estimated that 459,000 people had dementia in 2020 [2], with over half of people living in permanent residential aged care living with dementia [3]. Dementia prevention has been identified as a health priority due to the high global economic burden, estimated to be as high as US$ 1.3 trillion in 2019 [1]. Physical inactivity has been identified as one of the modifiable risk factors for dementia, with preventative individually tailored interventions incorporating physical activity being one approach with growing evidence of being able to reduce the risk of developing dementia [4].

Providing physical and social activities as part of care in aged care facilities is important for the maintenance of health and independence. In particular, physical activity programs that target daily living functional mobility (e.g., sit to stand, transfer movements) can be effective in improving mobility limitations and participation in older people with dementia [5]. Engagement in meaningful and enjoyable activities has also been shown to result in positive health outcomes for people living with dementia [6, 7].

The Seniors Exercise Park consists of outdoor exercise equipment that has been designed for older people and has been installed in several public spaces, parks, retirement villages and aged care facilities in Australia. Usage of the Seniors Exercise Park led to improvement in physical and social health for older people in the community without cognitive impairment [8–10] and for those with balance dysfunction [11]. The unique aspects of the Seniors Exercise Park are that they provide a fun and physically challenging environment to support active engagement in movements aimed at improving balance (e.g., unstable walking bridge, narrow walking beam), flexibility (e.g., shoulder range of movement), strength and functional mobility (e.g., sit to stand, stairs) [12]. Importantly, older people reported enjoyment and increased social interaction during their participation in physical activity program [13–15]. The growing evidence and popularity of the physical activity program utilising the Seniors Exercise Park (‘the Exercise interveNtion outdoor proJect in the cOmmunitY’**;** ENJOY program) highlighted this novel approach as an important public health infrastructure investment in promoting physical activity for older people without cognitive impairment [9, 15]. The present study, the ENJOY program for independence in dementia, aimed to pilot this innovative outdoor physical activity and social program with people living with dementia in a residential aged-care setting. We aimed to examine the feasibility of delivering the physical activity program for people with mild to moderate dementia, in terms of the exercise program structure, its safety, and supervision needs. In addition, the physical, social, health and cognitive benefits of participation in the ENJOY Seniors Exercise Park program were examined.

## Methods 

All procedures involved in this trial were conducted in compliance with the National Statement on Ethical Human Research and the Australian Code for the Responsible Conduct of Research. Ethical approval was obtained from the Melbourne Health Ethics Committee, Melbourne Australia (HREC/61926/MH-2020). The study was designed according to the Consolidated Standard of Reporting Trials (CONSORT) guidelines and publications associated with the trial are reported according to the CONSORT 2010 Statement [16, 17]. All participants signed an informed consent form prior to participation. Where a participant didn’t have the capacity to consent, a nominated representative signed an informed consent form, along with assent from the participant.

## Design and setting

This study was a feasibility pilot randomised controlled trial with pre-post evaluation. Full details of study design and study procedures and assessments are provided in the protocol paper [18]. In brief, older people aged 60 years and over who resided in Leith Park aged-care facility in Melbourne and who experienced symptoms (such as memory loss) consistent with a diagnosis of dementia and/or had a formal diagnosis of dementia were recruited. Participants were randomised to either an exercise intervention group (Seniors Exercise Park program) or to a control group. The Seniors Exercise Park was installed in 2019.

The ENJOY Seniors Exercise Park program is a supervised structured 12-week exercise program with incremental increase in the number and / or intensity of exercises and exercise duration throughout the 12-weeks that has been previously delivered to older people with no cognitive impairment [19]. Participants from the intervention group underwent the ENJOY program, followed by a 12-week maintenance phase (unstructured independent exercise under supervision). Participants from the control group were given the opportunity to participate in organised recreation and leisure-based group activities that were part of the aged-care facility’s activities. All participants were assessed at baseline, 12-weeks and 24-weeks.

### Study population

#### Inclusion criteria

Older people from the Leith Park Aged-Care Facility were recruited if they met the following inclusion criteria: 1) aged 60 years or older and residing at the aged-care facility 2) had been diagnosed with mild to moderate dementia and a screening test score of Standardised Mini-Mental State Examination (sMMSE ˃10), OR had cognitive symptoms that, in the opinion of the experienced staff, were consistent with mild to moderate dementia, and a screening test score of sMMSE ˃10 [20], 3) were able to stand by themselves with or without hand support; 4) were able to walk (with or without a walking aid) without physical assistance from staff and, 5) were able to follow simple exercise instructions.

#### Exclusion criteria

Older adults were excluded from this study if they: 1) were unable to stand by themselves with or without hand support; (2) were unable to walk without physical assistance from staff (with or without walking aid); (3) were unable to comprehend simple instructions during the exercise program (determined by the intervention health professional within the first two classes, based on their observation of each participant); (4) had severe dementia (score on the sMMSE ≤ 10) [20]; (5) scored on the sMMSE > 24; and/or (6) had other terminal or unstable illness or chronic conditions, or any documented medical condition or physical impairment that was deemed by their medical practitioner to contraindicate their inclusion.

### Recruitment and consent process

The aged care residents and their family members were informed about the study by the aged-care staff via verbal communication and newsletter. Potential participants meeting the inclusion criteria were identified by aged-care staff. Those who were diagnosed with mild to moderate dementia or who experienced symptoms (such as memory loss) consistent with a diagnosis of dementia (with a sMMSE score of ˃10) were given information about the study and were screened for eligibility. Details of eligible and interested participants were then provided to the research staff, who followed up with a phone call/email (to the family member/nominated representative) or visited the aged-care facility to further ascertain the resident’s interest in participating, and their capacity to consent. During that visit, the researchers provided the resident with written information. If it was deemed that the participant had the capacity to consent and agreed to participate, baseline testing took place or an appointment was made for the baseline testing. If it was determined that the resident didn’t have capacity to consent but could participate in some or all parts of the project, the research team contacted the nominated representative of the resident to sign an informed consent form along with assent from the resident.

Research staff were trained by a registered neuropsychologist (AG) to determine participant’s capacity to consent. The following indications were considered for capacity to consent: the person understood the information about the project, retained the information to the extent necessary to decide to participate, was able to use or weigh the information about the project in the process of deciding to participate, and could communicate the decision in some way.

### Randomisation

Randomisation took place after completion of baseline testing. Participants were randomly allocated (1:1) to one of the following groups: (1) control group or (2) Seniors Exercise Park program group. Randomisation was stratified by dementia severity (moderate or mild) based on sMMSE score (moderate: sMMSE ˃10 but ˂20; mild: sMMSE ≥ 20 but ≤ 24) [20]. Block randomization was undertaken using opaque envelopes, so that blocks of 6–8 participants (3–4 for intervention group and 3–4 for control group) were randomised at a time. Assessors, aged care staff and participants were not blinded to their respective group allocation. For those who were randomised to the exercise intervention group, a medical clearance from the General Practitioner on-site was sought prior to commencing participation in the exercise program.

### Procedure

The following information was collected at baseline: demographic characteristics (age, sex), anthropometric measures (height and weight), previous medical history, current medication usage, socioeconomic and cultural background information (e.g. employment, level of education, country of birth, years of residency in Australia) and falls history (number of falls in the past 12 months). To cross-check and to optimise accurate data collection, information about medical history, medication usage, dementia diagnosis, and falls history was also extracted from the participant’s medical record kept onsite.

Participants underwent a comprehensive suite of measures at baseline, 12 and 24-weeks that included physical function (strength, balance, functional mobility) tests, psychosocial (quality of life, loneliness, depression) questionnaires, falls risk assessment and falls history assessments (details below).

### Assessments

#### Primary outcome

The primary outcomes included feasibility, safety, and supervisory needs associated with the program:

##### Feasibility

The following criteria were used to determine the feasibility of the program: recruitment (% approached who agreed to participate), completion of the intervention (retention), attendance, overall adherence, dropout rate, adverse events (falls, muscle/joint pain), any modification in the exercise program delivery (optimisation of program structure) and supervision needs of the participants. High variability (25.5%-84%) has been reported in studies for adherence to exercise intervention in people with dementia in aged-care settings [21]. An estimated participation rate of ≥ 70% of the prescribed number of exercise sessions (24) during the 12-week structured exercise program, and ≥ 60% adherence for the overall 24-week exercise program were considered acceptable. Retention of 85% of the sample was targeted (with estimate 15% drop out) at 24-weeks.

Safety was assessed as follows: any falls (defined as an event when the participant ‘inadvertently comes to rest on the ground, floor or other lower level’ (WHO Global Report on Falls Prevention in Older Age [22])) that occurred during the exercise delivery sessions, any adverse events (joint/muscle pain during the exercise, or as a result of the exercise sessions), serious adverse events (any report of difficulty breathing, new or unrelenting chest pain, or acute changes in the level of consciousness requiring a medical emergency).

Physical activity program optimisation / adaptations to the program structure were assessed by documenting any modifications to the method of delivery of the overall exercise program and/or individual exercise sessions (e.g. exercise length, session duration) due to safety concerns, and other reasons (e.g. mental or physical fatigue). During the maintenance phase (after completion of the formal supervised component, from 12 to 24-weeks post baseline), the exercise instructor/staff supervised the residents and encouraged them to be more independent and to gradually self-manage the way they used the equipment and exercise. Exercise behaviours of each participant were monitored and recorded by the exercise instructor and/or the aged care staff. Field notes (post session reflection notes) were completed by the research staff noting down any variations of the program, participant’s behaviour and any other relevant aspects identified by the staff.

Supervision requirements were assessed via documenting the supervision ratio, which was reassessed regularly and adjusted if needed, based on discussion with the staff involved in the program delivery around safety and the need to guide and support residents. Due to the potential complexity of running an exercise program with people living with cognitive impairment and potential mobility problems (e.g., usage of walking aid), we aimed initially for one qualified instructor (Exercise Physiologist/Physiotherapist) and another supervisor (from the aged-care staff) with 3–4 participants for each supervised outdoors exercise park session.

#### Secondary outcomes

The following suite of measures were collected as part of the secondary outcomes: physical function (strength, balance, functional mobility), psychosocial (quality of life, depression, loneliness), cognitive, and falls risk and falls history domains. Physical activity participation, engagement, social interaction, mood and enjoyment data were also collected. Assessment timepoints are provided in Table 1.

**Table 1:** Timeline of assessments and data collection

Assessment	Baseline	12 weeks	24 weeks	During exercise intervention
Informed consent, demographic information	✓			
Physical function measures	✓	✓	✓	
Cognitive and health related quality of life	✓	✓	✓	
Participants feedback survey		✓		
Mood				✓At the beginning of each session -completed by participant
Enjoyment				✓At the end of each session - completed by participant
Motivation to participate				✓At each session - completed by staff
GOME: Group				✓At each session - completed by staff
GOME: Individual				✓At each session - completed by staff
General motivation to go				✓At 4 time points: week 1, week 6, week 12, week 24 - completed by staff
Field notes (post session reflection notes)				✓At each session throughout the intervention period as needed - completed by staff

#### Physical function measures

Physiological measures of strength, balance and functional mobility were assessed using the following validated tests as detailed in the protocol paper [18]:

(i) Functional lower limb muscle strength using the five times sit to stand test [23]. The time taken to complete the task was measured (seconds).
(ii) Exercise tolerance and functional mobility using the two-minute walk test [24]. The distance covered with usual walking aid during two minutes was recorded (metres).
(iii) Dynamic balance using the Step test [25]. The number of steps (7.5-cm-high step) completed in a 15-s period for each leg was recorded. The sum of steps of the two legs was used in the analysis.
(iv) Walking speed—using the Four Meters Walk test [26]. Participants were asked to walk four meters at their comfortable walking pace and with their usual walking aid. Gait speed was reported by dividing the distance by time (in seconds) it took to walk four meters.

#### Psychosocial, cognitive and quality of life health outcomes

Several valid, reliable instruments (validated in older people and those with dementia) for cognitive screening, quality of life, socialisation, daily activity engagement and depression were used, as detailed in the protocol paper [18].

(i) *The Montreal Cognitive Assessment (MoCA)* is a brief 30-question cognitive screening test that assesses several cognitive domains [27]. The total possible score is 30 points; a score of 26 or above is considered normal. Staff assessing MoCA underwent the required training.
(ii) *Health-related quality of life* was assessed using two instruments: the Quality of Life in Alzheimer's disease scale (QoL-AD) and the EQ-5D-5L [28, 29]. QoL-AD total scores range from 13 to 52, with higher score represents better quality of life. For the EQ-5D-5L, the Visual Analog Scale (VAS) 0–100) is reported where higher score represents better health.
(iii) *Fear of falls* was assessed using the Iconographical Falls Efficacy Scale, a valid and reliable scale that assesses fear of falling in older people with cognitive impairment [30]. Score ranges between 10–40 where higher score represents greater concerns about falling.
(iv) *Loneliness* was assessed using the UCLA 3-Item Loneliness Scale [31, 32] with scores range from 3 to 9 (higher scores indicating greater feelings of loneliness).
(v) *Depression* was assessed using the short version Geriatric Depression Scale (GDS-15) [33]. A score of 0 to 5 is considered normal and a score greater than 5 suggests depressive symptoms.
(vi) *Social isolation and social support* was assessed using the short version 6 items Lubben Social Network Scale (LSNS-6) [34]. The score ranges between 0 and 30 where higher scores indicate more social engagement.
(vii) *Engagement with daily activity* was assessed by aged-care staff using the Pool Activity Level (PAL), a widely used, validated, measure of engagement with activity for older people with dementia. The PAL rates the ability to plan and perform nine common daily activities [35]. Total score ranges between 9–36, with higher score represents higher activity level.

#### Falls risk assessment

(i) *Falls risk was assessed using the Falls Risk Assessment Tool (FRAT)* [36]. The FRAT is a 4-item falls-risk screening tool for sub-acute and residential care: with risk factors scored to reflect graded risk of low (score 5–11), moderate (score 12–15) and severe risk (score 16–20).

#### Perceptions and feedback of the physical activity program – from aged-care staff and from participants

During the physical activity program delivery.

### Measures completed by residents/participants

1. Mood was assessed at the beginning of each session using a five-point Likert scale using a visual smiley face card (from sad, depressed, down to very happy high).
2. Enjoyment was assessed at the end of the exercise session. Participants rated their level of enjoyment on a five-point Likert scale using a visual smiley face card ( e.g., not at all, neutral, had fun).

### Measures completed by staff (research instructor, facility staff):

1. Motivation to participate—Participants’ motivation was assessed by the exercise supervisor at each exercise session based on their observation and interpretation of participants’ expression, verbal prompt and body language using a five-point Likert scale, from no motivation to very high motivation [37].
2. Group observational measurement of engagement (GOME)—was used to evaluate engagement in the physical and social activity and includes engagement on an individual and group levels using a Likert scale [38].

The GOME group level engagement included record of number of participants in the group, and positive and negative interactions using a 6-point Likert scale.

3. Wandering behavior—Any wandering behaviour (walking off) around the park area, was documented by the staff.
4. General motivation and engagement behaviour at selected time points throughout the program—Motivation to go to the activity was assessed at 4 time points: at week one of the program, halfway through (week 6) and at the end (week 12), and at the completion of the maintenance phase (week 24). Motivation was assessed using a five-point Likert scale from very negative (0, never wants to or usually does not want to go to sessions, despite motivating attempts) to very positive (score 4) [37].

### Overall feedback from both residents/participants and staff

Overall experience and feedback about the exercise program was collected at the completion of the 12-week exercise intervention from residents/participants and staff. Residents feedback was collected at the completion of each exercise group using a survey (a questionnaire incorporating open ended and rating scale questions).

### Exercise park intervention

#### Intervention group: 12-week structured supervised exercise program

**Fig. 1:**
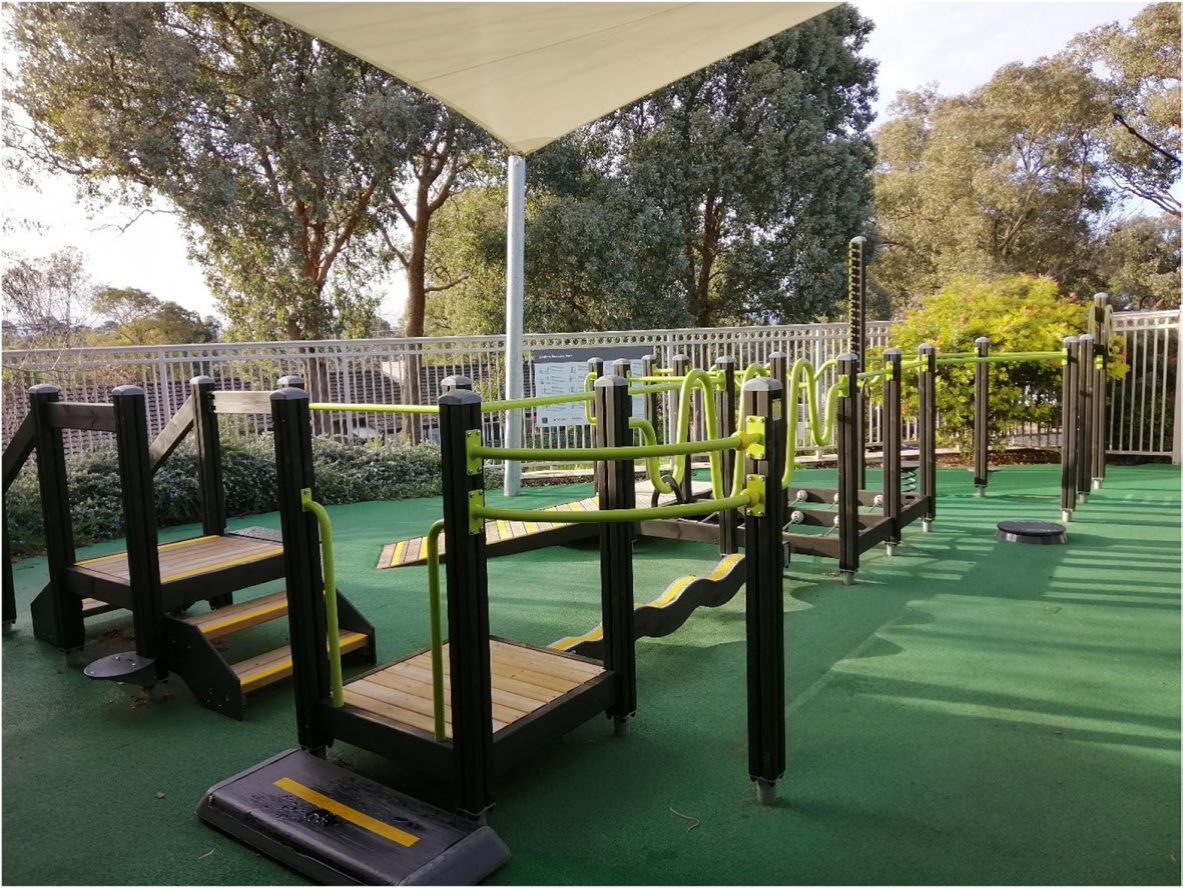
The Seniors Exercise Park at the Leith Park aged care facility in Melbourne, Australia

Participants underwent a 12-week supervised exercise intervention program twice weekly using the Seniors Exercise Park. The exercise park equipment is outdoor playground equipment comprising multiple equipment stations that target a specific function or movement (upper and lower limb), range of movement, static and dynamic balance, or functional movement such as walking up/down stairs and sit to stand (Fig. 1) [12, 19]. Examples of the exercises can be found here https://youtu.be/PaYuCMtnlYk. Each class was approximately 1 to 1.5 h duration, and was supervised by a qualified Accredited Exercise Physiologist with the assistance of the Diversional Therapist (aged care staff). Each session consisted of 5–7 min warm-up exercises, followed by 45–75 min on the equipment stations. The exercise classes included 3–4 participants and was circuit-based. Morning tea (light refreshment) was organised following the exercise sessions. The level of the exercise difficulty was tailored to the capabilities of each participant with the primary consideration of safety, with adjustment of the exercises difficulty throughout the program based on the individual participant progression. Details on the structure and progression of the exercises are provided in the protocol paper [18] and similar to our previous studies [12, 19]. Variations and or modifications that were made to the program were noted. Adaptations and/or modifications to the program followed the updated Framework for Reporting Adaptations and Modifications-Expanded (FRAME) guidelines [39].

#### Exercise uptake and physical activity maintenance (maintenance phase: 12–24 weeks post baseline)

After completion of the 12-week program, scheduled sessions were available for participants to access and use the Seniors Exercise Park under supervision (aged-care/ Diversional Therapist staff from the facility). To facilitate independence and empowerment, participants were encouraged to exercise independently (supervised but unstructured sessions). Participants from intervention group also participated in other leisure/exercise activities (that didn’t clash with the scheduled sessions) provided as part of usual care at the facility.

### Control group

Participants from the control group participated in any organised recreation and leisure-based group activities that were run by the Diversional Therapist staff at the aged-care facility. As the exercise intervention provided did not replace any other physical activities, control participants were not withheld from receiving physical activity programs or any related one-on-one physiotherapist sessions.

## Statistical methods

### Sample size estimation and justification

As this was a feasibility study, we aimed to recruit 12 participants for each group. This was based on the available number of residents at Leith Park residential aged-care facility, with an estimation that approximately 37–40 residents living with dementia were at the facility at the time of study design. A targeted total number of 24 participants (20 participants completing the study allowing for ~ 15% drop out rate) seemed feasible to allow completion of the trial over the 24-week period. Our previous trial had 11% drop out from the exercise intervention group [10]. The targeted number would also be sufficient to provide preliminary results about the feasibility and safety of the exercise. The sample size was not powered to detect significant changes in the secondary measures. Non parametric test was used to compare the differences in the proportion of fallers (falls not related to the exercise program) between the groups at baseline and at the 24-weeks follow up.

### Statistical analysis

The data for the feasibility and safety components of the study were analysed as follows (mean, standard deviation and proportion): proportion of participants approached to participate and commenced the program, percentage of participants who completed the intervention, overall percentage of sessions attended, number of participants who dropped out, number of falls that occurred during the exercise sessions, frequency of muscle/joint pain during or after the exercise sessions, and number of serious adverse events requiring medical attention. Modifications made to the exercise program were also recorded.

To determine trends of effectiveness, repeated measures ANOVA with factors of intervention (Seniors Exercise Park program, control) and time (pre-post intervention and follow-up) were used for the secondary outcomes (cognitive, physical function, quality of life and social measures) to assess the changes within and between groups over time (pre/post). Effect size, Partial Eta Squared, (${\eta }_{p}^{2}$) from SPSS for the group by time interaction was used and reported to determine effect size as follows: ${\eta }_{p}^{2}$ values greater than 0.14 were considered a large and significant effect size whereas 0.01 and 0.06 were considered small and medium effect size, respectively [40].

## Results

### Feasibility outcomes

**Fig. 2:**
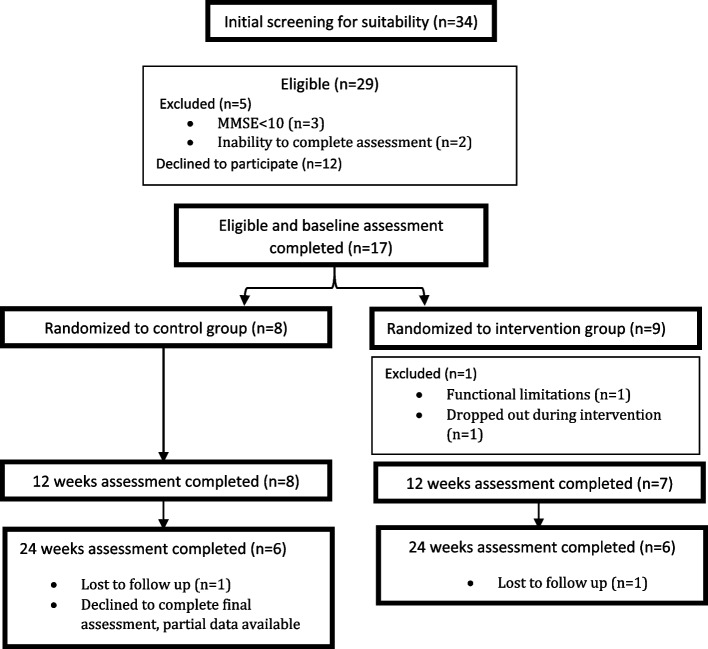
Flow diagram of recruitment and drop out

Recruitment took place between Jan 2021 to June 2022 with intermediate breaks and delays due to COVID-19 restrictions and lockdowns. Thirty-four residents were screened and identified as potentially suitable, five residents were excluded (three due to MMSE < 10 and two due to inability to complete assessment). Further details are provided in Fig. 2. A total of 29 residents met the eligibility criteria after screening. Of these, 12 declined to participate, and 17 completed baseline, indicating a recruitment rate of 58.6% (17/29). Seven intervention participants (87.5%) completed the entire 12-week program, with 75% retention of these at the 24-week follow up (one lost to follow up during the maintenance phase, between 12–24 weeks). Eight people were in the control group.

The demographics of the control and intervention group are presented in Table 2. The average age was 87.5 ± 3.0 (87.5% women) and 83.3 ± 7.5 (87.5% women) years for the control and intervention groups respectively. The most common medical conditions reported were arthritis/joint pain (87.5% control, 62.5% intervention), hypertension (62.5% control, 75.0% intervention), hearing impairments (62.5% control, 62.5% intervention) and incontinence (62.5% for both control and intervention). All participants took medications, with pain relieving, anti-hypertensives and anti-depressants being the most common medications (Table 2).

**Table 2:** Demographic characteristics of the control and intervention groups

Variables	Control *n* = 8	Intervention *n* = 8
Age (yrs), mean ± SD	87.5 ± 3.0	83.3 ± 7.5
Females (%)	7 (87.5)	7 (87.5)
Height (m), mean ± SD	1.5 ± 0.07	1.5 ± 0.08
Weight (kg), mean ± SD	70.3 ± 25.0	68.8 ± 15.4
BMI (kg/m^2^), mean ± SD	27.4 ± 7.4	27.4 ± 5.0
sMMSE	19.8 ± 4.7	20.2 ± 2.2
Number fallers in preceding 12 months (%)	2 (25%)	3 (37.5%)
Smoking	0	0
**Medical conditions and Musculoskeletal conditions n (%)**		
Dementia type diagnosis:		
Alzheimer disease	1 (12.5)	5 (62.5)
Dementia – type not specified	2 (25)	2 (25.0)
Mixed dementia	1 (12.5)	–
Vascular dementia	–	1 (12.5)
Symptoms of dementia (no formal diagnosis)	4 (50)	–
Arthritis/joint pain (Osteoarthritis/Rheumatoid Arthritis/undiagnosed pain)	7 (87.5)	5 (62.5)
Hypertension	5 (62.5)	6 (75.0)
Hearing impairments	5 (62.5)	5 (62.5)
Incontinence	5 (62.5)	5 (62.5)
Hypercholesterolemia	3 (37.5)	4 (50.0)
Depression/anxiety	4 (50)	3 (37.5)
Osteoporosis	3 (37.5)	2 (25.0)
Cardiovascular conditions	1 (12.5)	2 (25.0)
Diabetes mellitus	1 (12.5)	0
Other metabolic conditions (Kidney/Thyroid Disorder)	1 (12.5)	2 (25.0)
**Medication usage and type, n (%)**		
Taking medications	8 (100)	8 (100)
Median number of medications (Interquartile Range)	9 (3.7)	8 (6.2)
Pain relieving medications	6 (75)	7 (87.5)
Hypertensive medications	6 (75)	3 (37.5)
Anti-depressant medications	6 (75)	2 (25.0)
Cholesterol-lowering medications	1 (12.5)	3 (37.5)
Blood Thinners	4 (50)	1 (12.5)
Respiratory medications	0	1 (12.5)
Glucose lowering medications	1 (12.5)	0
Anti-inflammatory medications	1 (12.5)	2 (25.0)
Hormonal therapy medications	2 (25)	1 (12.5)
**Socio-economic and education status**		
Education level—Secondary school or below (%)	3 (37.5) 1 (12.5) unsure	3 (37.5)
First generation migrants (born overseas)	3 (37.5); Europe 1 (12.5) unsure	2 (25.0); Europe
English first language	8 (100)	8 (100)
**Marital status**		
Married/spouse	-	2 (28.5)
Widowed	5 (62.5)	5 (62.5)
Single/divorced/separated	3 (37.5)	1 (14.2)

*sMMSE* Standardised Mini-Mental State Examination; BMI = body mass index

Adherence to the 12-week structured program and the maintenance phase for the intervention group was 99.4% and 69.4% respectively, with an adherence of 84.3% for the overall 24-week intervention period. Reasons for lack of attendance were as follows: being sick with COVID-19 or in isolation, weather too hot/cold, other commitment (e.g. partner taking participant out), although in some instances this information was not available especially during the COVID-19 restrictions. During the conduct of the study, the logistics of recruitment and exercise program delivery included running it in two small groups with staggered commencement of participants. This enabled ongoing recruitment and adjustment due to COVID-19 restrictions. It also allowed providing top-up sessions due to cancellation of sessions.

#### Safety

No falls occurred during the exercise sessions that were part of the 24-week exercise program. There were 13 instances where participants reported pain during the exercise program sessions which were mainly due to pre-existing conditions (joint pain). No other events were reported.

Impact of COVID19 lockdown and closure – several sessions were cancelled due to COVID-19 restrictions (no access to the aged care facility and travel restrictions) or the aged care staff ran the session in a reduced capacity (research staff not allowed to access the aged care facility) and for a shorter session period. There were 21 instances of session cancellations across the study duration, 10.4% (15 sessions) due to lockdown associated with COVID19 restrictions and 4.1% (6 sessions) due to weather (heavy rain).

#### Supervision needs/ratio

The exercise program was facilitated mostly with two staff (a qualified exercise instructor and an aged care staff member—a Diversional Therapist from the Lifestyle team) with a group of three to four participants. There were instances where student volunteers assisted in the delivery of the program enabling one resident to one staff/volunteer. In this study, a ratio of two residents to one staff was practically possible, although a ratio of one to one seemed ideal. During the maintenance phase where participants were used to the exercise and exercised more independently, one staff to two residents was sufficient.

#### Physical activity program optimisation / adaptations to the program structure

In the initial three months, all participants needed constant instructions with ongoing repetition of the information and demonstration. As sessions progressed, participants became less reliant on instructions and mainly just needed correction of technique and adjustment of progression. The circuit-based approach, with incremental increase in number of exercises and duration of the exercise program was able to be followed. However, adjustments in terms of communication and motivation were needed to suit different individuals’ personality, needs and preferences. Progression of the exercises as per exercise program protocol was the most challenging aspect of delivering the program due to participants experiencing difficulties retaining memories of the previous level / session activity, due to their cognitive impairment. Ongoing verbal reinforcement (reassurance to build confidence), the usage of external videos of residents performing the exercise, and exercise sheets with details of repetitions assisted some residents to successfully complete the program. The usage of small cards (with illustrations and information about the exercises) fitted on each exercise station were useful for some residents while for others this was distracting and confusing. A summary of the modifications for the exercise program is provided in Table 3 using the FRAME [39].

**Table 3:** Summary of the modifications made for the exercise intervention program using the FRAME

	**Process**		
**When did the modifications occur?**	**What is modified?**	**At what level of delivery? (from whom the modifications made)**	**What is the nature of the content modification?**
-During piloting	Contextual delivery • Methods of communication • Usage of external aids • Supervision ratio	Targeted intervention group – participating residents with mild to moderate dementia	• Tailoring to individual resident • Adjusting staff ratio for safety • Ensuring quiet environment • Training in small groups • Using verbal cues with simple, short and concise instructions
**Were adaptations planned?** -Planned/reactive – reactive adaptation during intervention delivery			
**Who participated in the decisions to modify?** -Diversional therapist (aged care staff) -Exercise instructor -Lead researcher		**Contextual modifications made to which of the following?** -Personal	
			**Relationship fidelity/core elements** Fidelity consistent/core elements were preserved
**Reason**			
**What was the goal of modifications?** To improve the participation and experience of people living with dementia in use of the Seniors Exercise Park physical activity program		**What influenced the decision?** **The recipient:** -Cognitive ability of residents to retain information -Encourage motivation for participation	

##### Participants’ perceptions, feedback, and engagement

Average enjoyment recorded across all exercise sessions was high (3.6 ± 0.4, scale range 0–4), with average ‘happy’ positive mood (2.9 ± 0.6, scale range 0–4). Similarly, high motivation to participate was reported (3.0 ± 0.4, scale range 0–4), with high attendance duration, engagement and positive attitude and positive group interaction (Table 4). At the completion of the 12-weeks exercise program, participants reported that they enjoyed the program (100% agree/strongly agree), with 85.7% reporting being satisfied with the outcome of the program. Participants reported to enjoy the exercises (85.7%), followed by being outdoors (71.4%) and the social aspects of the program (71.4%), Table 4.

**Table 4:** Enjoyment, mood, engagement and feedback of the intervention group during the exercise session and at 12-weeks follow up (or otherwise indicated in the Table)

Measure	Intervention group
Motivation to participate (range 0–4)	3.0 ± 0.4
Enjoyment (range 0–4)	3.6 ± 0.4
Mood (range 0–4)	2.9 ± 0.6
^a^Motivation to go to the activity (range 0–4)	3.1 ± 0.4
**GOME Individual (average of all training sessions)**	
Attendance Duration (range 0–6)	5.8 ± 0.1
Engagement (range 0–5)	4.8 ± 0.1
Active participation (range 0–4)	3.6 ± 0.1
Attitude (range 0–7)	5.7 ± 0.3
Sleep like symptoms (range 0–6)	(0.005 ± 0.01)
**GOME Group (average of all training sessions)**	
Positive interaction (range 0–5)	3.7 ± 0.1
Negative interaction (range 0–5)	0.02 ± 0.03
**Feedback survey (at 12 weeks)**	
I enjoyed participation in the exercise program	Agree/Strongly agree 7 (100%)
I am satisfied with the outcome of the exercise program (my health/physical function improved)	Agree/Strongly agree 6 (85.7%)
What did you enjoy the most n (%)	
The Exercises	6 (85.7)
Being outdoors	5 (71.4)
Socialisation	5 (71.4)
Refreshment	4 (57.1)
Supervision	3 (42.8)

*GOME* Group observational measurement of engagement

^a^measured at week 1, 6, 12, 24

##### Secondary outcomes

There were no significant differences (*p* ≥ 0.05, small effect size) for group by time interaction in any of the physical, cognitive, and health outcomes, Table 5. Similarly, no significant differences were detected between any of the time points irrespective of the groups. Most outcome measures were mainly unchanged across all time points for both groups; although slight reduction in self-reported quality of life was reported for the control group and slight improvement in the step test was reported for the intervention group, although not significant.

**Table 5:** Secondary outcomes of both groups at baseline, 12 and 24-weeks follow up (values reported mean ± SD)

**Measure**	**Control *n* = 8**			**Intervention *n* = 7**			**p value group by time interaction**	${n}_{p}^{2}$**for group by time interaction**	***p* value between groups**	***P* value between timepoints**
	Baseline	12 weeks	24 weeks	Baseline	12 weeks	24 weeks				
Physical function										
Sit to stand	19.7 ± 8.3	21.5 ± 8.9	21.0 ± 1.6	17.5 ± 5.9	16.7 ± 6.2	16.5 ± 4.7	0.8	0.01^c^	0.07	0.9
Two-minute walk (m)	67.6 ± 16.8	72.5 ± 15.0	64.6 ± 15.5	107.7 ± 20.0	105.2 ± 16.9	105.6 ± 18.6	0.7	0.01^c^	˂0.01*	0.8
4 Meter Walking speed (m/s)	0.7 ± 0.1	0.7 ± 0.2	0.7 ± 0.1	1.1 ± 0.3	1.0 ± 2.1	1.0 ± 0.1	0.7	0.02^c^	˂0.01*	0.7
^d^Step test	16.5 ± 4.1	18.0 ± 5.8	15.1 ± 4.5	18.2 ± 4.3	20.8 ± 3.8	21.0 ± 5.7	0.5	0.03^c^	0.02**	0.5
Health related quality of life										
Cognitive assessment (MOCA)	11.5 ± 6.3	12 ± 6.7	8.8 ± 6.1	15.1 ± 3.2	13.5 ± 2.5	12.3 ± 4.2	0.8	0.01^c^	0.08	0.3
Quality of life (QoL-AD)	37.5 ± 7.8	39.2 ± 8.9	35.0 ± 7.4	36.7 ± 6.8	35.0 ± 5.2	35.5 ± 7.3	0.6	0.02^c^	0.5	0.7
Quality of life (EQ-5D-5L -VAS)	77.0 ± 16.8	83.7 ± 12.7	65.17 ± 25.5	82.8 ± 16.0	79.2 ± 17.1	79.0 ± 20.6	0.4	0.04^c^	0.3	0.3
Fear of falls IFES	18.5 ± 5.7	19.5 ± 8.4	19.3 ± 7.3	17.5 ± 4.6	17.2 ± 4.6	18.1 ± 5.4	0.9	0.02^c^	0.4	0.9
Pool Activity Level (PAL)	30.0 ± 4.1	31.1 ± 3.4	29.3 ± 4.4	30.2 ± 1.8	29.4 ± 2.6	28.8 ± 3.0	0.7	0.01^c^	0.5	0.6
Loneliness UCLA 3	4.3 ± 1.9	4.3 ± 1.4	5.3 ± 1.7	4.7 ± 1.6	5.1 ± 1.7	4.3 ± 1.3	0.3	0.05^c^	0.9	0.8
Depression (GDS-15)	3.5 ± 2.6	4.2 ± 3.5	4.5 ± 3.2	3.8 ± 2.4	2.2 ± 1.2	4.1 ± 2.9	0.5	0.03^c^	0.4	0.6
Falls risk assessment (FRAT)	12.2 ± 1.7	12.8 ± 2.2	13.6 ± 3.1	11.7 ± 3.4	12.0 ± 3.6	12.8 ± 2.4	0.9	0.001^c^	0.3	0.5
Social isolation (LSNS6)	14.5 ± 7.3	11.0 ± 6.8	13.0 ± 4.5	7.4 ± 6.4	7.0 ± 7.6	6.5 ± 5.0	0.7	0.01^c^	˂ 0.01**	0.7

^*^Significant at *p* < .05 between groups at baseline; 12-weeks post, and 24-weeks post

^**^Significant at *p* < .05 between groups at 24 weeks post

^a^Large effect size

^b^Medium effect size

^c^Small effect size

^d^The score is the sum of both limbs

Irrespective of time, significant differences between the groups were detected for the two-minute walk and 4 min walking speed physical tests (*p* ˂0.01), with the intervention group having greater function at baseline; 12-weeks post, and 24-weeks post. A significant difference was also detected for the intervention group in the step test at the 24-weeks post (*p* = 0.02), with the intervention group performing better. Significantly greater social isolation (*p* ˂0.01) was identified for the intervention group at the 24-weeks follow up, although it was noted that social isolation was relatively low across all timepoints, Table 5.

No differences were found in the proportion of fallers in the preceding year between the groups (25% for control and 37.5% intervention, *p* ≥ 0.05) at the baseline. Similarly, the proportion of fallers reported in the prospective 24-weeks follow up was similar between the groups (42.8% and 28.5%, *p* ≥ 0.05) for the control and intervention groups respectively.

## Discussion

In this pilot feasibility study, an innovative approach was examined as a way to engage people living with dementia in a physical and social activity program (the ENJOY program) using outdoors Seniors Exercise Park equipment. While this approach has been quite successful in people living in the community demonstrating physical, social and health benefits [8–11], this has never been tested with people with cognitive impairment. We encountered substantial challenges conducting the trial during the COVID-19 pandemic, which impacted on recruitment rate, access to the aged care facility, and continuity of the exercise program delivery. A modest recruitment rate of 58.6% was reported, with 87.5% and 75% retention at 12-weeks and 24-weeks, respectively. We previously reported 80.1% retention for a longer period of participation (9 months), although that was in people living in the community with no cognitive impairment [9]. Low recruitment and retention of people living with dementia is common in research trials [41], with greater time and effort required to recruit this relatively hard-to-reach group. In terms of adherence, we reported relatively high attendance, especially in the initial 12-weeks (99.4%), which then reduced to 69.4% during the maintenance phase with an overall adherence of 84.3% for the 24-weeks intervention period. Adherence to physical activity programs (attendance rate) varies between previous studies [21], and several other factors have been identified in previous studies as barriers for group exercise attendance in aged care facilities including: bio-medical reasons including mental wellbeing and physical ability (e.g., illness, anxiety and agitation, depression, increased disability), relationship dynamics (e.g., disagreement with group members), and socioeconomic reasons [21]. In the present study, the research team worked closely with the aged care staff to reduce some of these barriers by using strategies that included knowing the residents well, leveraging the strong inter-relationships between residents, and involving staff in the exercise delivery (preparing residents, taking them to the park and assisting with the exercise program delivery). In addition, relationship dynamics between the exercise instructor, staff and group were well managed by anticipating challenges and learning what residents responded positively to.

There is growing evidence for the importance of lifestyle factors such as social and physical activities in positively influencing on mental health, quality of life, and rate of cognitive decline in people living with dementia, including after diagnosis [42]. Results from the secondary outcome measures analysis demonstrated no significant improvements over time (12 or 24-weeks) for either the intervention or control group. While the sample size was likely underpowered to detect any significant changes, it is also noted that there was no worsening of any of the physical, social and health outcomes. It is also important to acknowledge that participants from the control group were not withheld from participating in other activities provided as part of the care of the aged care facility, and indeed took part in other physical and social activities such as weekly fall-prevention and balance exercise sessions. This may explain in part the improvement seen in some of the physical function measures. However, despite the lack of significant changes in the secondary outcomes, the Seniors Exercise Park program was well received by residents with cognitive impairment, with reports of positive mood and enjoyment throughout the program as well as positive engagement and attitude. For example, feedback provided by the participants highlighted that the exercises, being outdoors and the socialisation were contributing factors for their enjoyment, which were similar to what has been reported by older people living in the community [15]. The unique aspect of the intervention combining specialised age-friendly equipment and exercising outdoors is important as being outdoors may have added psychological health benefits on mental wellbeing [43–45] and cognition [46]. The outdoor area where the Seniors Exercise Park is installed is an age-friendly space with greenery, seating area and tables, providing a pleasant area for residents, including having morning tea there following the training. In addition, the area is adjacent to the main entry to the facility but separate from other common areas, which was quiet to minimise distraction and sufficiently large to allow safe exercise in small groups.

While the study results are promising, sustainable usage of the equipment on an ongoing basis will require commitment from aged care staff to allow residents to continue to exercise in some capacity under suitable supervision. In addition, a follow up study will be required with a larger sample size to further identify potential social, physical, and health benefits of the ENJOY program for people living with dementia.

As part of the feasibility aspect of this study, we also examined the delivery of the ENJOY program and factors that needed adjustments to optimise usage of the equipment in this cohort. Close supervision was required in the initial 12-week program, which gradually reduced as the program progressed into the maintenance phase with a ratio of one staff to two residents being safe and practical. During the maintenance phase, participants seemed comfortable with the exercises and needed minimal instructions and guidance on how to perform the exercises. An alternative option to assist with supervision might include training family members and friends to support and supervise the person with dementia in using the Seniors Exercise Park, after a formal training period with therapists/staff. This might assist in reducing the resources/staffing required for longer term supervision and might also benefit the family members/friends if exercising together.

Progression of the exercises was the most challenging aspect of delivering the program as participants sometimes had difficulty recalling the level they were at from the last session. This created a challenge to the ENJOY program implementation as exercise progression is an important element of physical activity training which aims to increase overload over time to facilitate ongoing improvement. As the ENJOY Seniors Exercise Park program is a progressive exercise program, the ability of the participant to progress with the increasing difficulty of the exercises was often impacted by their cognitive impairment, particularly regarding their memory of their previous performance/level of difficulty. This, in turn, sometimes inadvertently impacted their confidence and willingness to attempt more challenging tasks. Adjustments of communication and usage of external aids were tested and individualised to ensure they were positively contributing and not distracting and confusing for individual residents. Overall, implementing strategies to assist with exercise progression is an important factor to consider for exercise prescription for people with cognitive impairment and those living with dementia.

Based on the present exercise program delivery experience and associated modifications, the following practical recommendations will enable safe and pleasant exercise sessions for people with cognitive impairment and those living with dementia: 1) ensure the environment is quiet and welcoming (training in small groups and maintaining distance where exercise participants cannot overhear conversations of others), 2) provide simple, short and concise instructions, 3) maintain a routine and use repetition (e.g., same morning routine for the exercise days, preparation), 4) be patient and allow sufficient time (several weeks) for participants to feel comfortable performing the exercise with ease, 5) minimise the instructions as the weeks progress, gradually shifting towards giving control to the individual to self-manage their own exercise routine, 6) personalise the communication techniques as every person responds differently to instructions and feedback (example strategies are knowing the resident, and knowing what they respond well to, and what they do not respond well to). A summary of these recommendations is provided in this educational video https://www.youtube.com/watch?v=cgqQjXlFN_0

People with dementia have lower physical activity levels than older people with no cognitive impairment [47], and people living in residential care (with or without dementia) have very low levels of physical activity [48]. Novel approaches to increase physical activity participation for residents in residential care, including those with dementia, are required. Increasing use of outdoor areas generally, including installing and using purpose built outdoor exercise equipment such as the Seniors Exercise Park utilised in this study, should be considered. Using practical learnings from this study can be valuable for future physical activity interventions for people living with dementia with or without the usage of the Seniors Exercise Park. Despite the challenging aspects of working with this group, physical activity provides important health benefits particularly for the maintenance of physical independence. The gradual ability of the residents to exercise more independently (e.g., reduced instructions from the trainer during the maintenance phase) was a positive indication for the retention of movements and improved mobility. Consequently, this study highlights the potential positive impact of a suitable well-designed built environment in residential aged-care facilities to improve the care provided for people living with dementia.

This study has several limitations. Firstly, the COVID-19 pandemic and associated restrictions and lockdowns resulted in recruitment challenges and a lower sample size to what was targeted. Illness or isolation of the residents due to COVID-19 also impacted attendance and may have prevented potential improvement. Although 12-weeks of consistent progressive exercise is commonly sufficient to produce improvement in physical mobility in older people without dementia, it is possible that a longer supervised program duration is required to create a significant change in physical or cognitive capacity in this population [49, 50]. Additionally, both assessors and participants were not blinded to their respective group allocation. While there are clear benefits from a research perspective to blind assessors, having the same staff undertake assessments and interventions was found to be important in reducing confusion and enabling relationships and trust to be built. There is ample evidence that the relationship between the staff and residents is an important factor in facilitating adherence and participation [21].

## Conclusion

The ENJOY Seniors Exercise Park physical activity program was safe and feasible to use for people living with mild to moderate dementia in an aged care facility. Increased enjoyment, positive attitude, and high levels of engagement were demonstrated, with high adherence to the exercise program. Adjustments to the physical activity program, in terms of communication, motivation and progression were needed to suit individual personality and characteristics. A fully powered study is now needed to assess the effectiveness of the program on social, physical and cognitive function for people living with dementia in residential aged care.

## Data Availability

The datasets generated and/or analysed during the current study are not publicly available due ethical constrains but are available from the corresponding author on reasonable request.

## Abbreviations

ENJOY: Exercise interveNtion outdoor proJect in the cOmmunitY
QoL-AD: Quality of Life in Alzheimer's disease scale
EQ-5D-5L: The 5-level EQ-5D
sMMSE: Standardised Mini-Mental State Examination
UCLA: University of California at Los Angeles Loneliness Scale
MoCA: Montreal Cognitive Assessment
GDS: Geriatric Depression Scale
PAL: Pool Activity Level
IFES: Iconographical Falls Efficacy Scale
FRAT: The Falls Risk Assessment Tool
GOME: Group Observational Measurement of Engagement
FRAME: Framework for Reporting Adaptations and Modifications-Expanded
